# Choline Protects the Heart from Doxorubicin-Induced Cardiotoxicity through Vagal Activation and Nrf2/HO-1 Pathway

**DOI:** 10.1155/2022/4740931

**Published:** 2022-04-05

**Authors:** Fuding Guo, Yueyi Wang, Jun Wang, Zhihao Liu, Yanqiu Lai, Zhen Zhou, Zihan Liu, Yuyang Zhou, Xiao Xu, Zeyan Li, Meng Wang, Fu Yu, Ruijie Hu, Liping Zhou, Hong Jiang

**Affiliations:** ^1^Department of Cardiology, Renmin Hospital of Wuhan University, Wuhan, China; ^2^Cardiac Autonomic Nervous System Research Centre of Wuhan University, Wuhan, China; ^3^Cardiovascular Research Institute, Wuhan University, Wuhan, China; ^4^Hubei Key Laboratory of Cardiology, Wuhan, China

## Abstract

Choline is a precursor of the major neurotransmitter acetylcholine and has been demonstrated beneficial in diverse models of cardiovascular disease. Here, we sought to verify that choline protects the heart from DOX-induced cardiotoxicity and the underlying mechanisms. The results showed that DOX treatment decreased left ventricular ejection fraction and fractional shortening and increased serum cardiac markers and myocardial fibrosis, which were alleviated by cotreatment with choline. DOX-induced cardiotoxicity was accompanied by increases in oxidative stress, inflammation, and apoptosis, which were rectified by choline cotreatment. Levels of nuclear factor erythroid 2-related factor 2 (Nrf2) and heme-oxygenase-1 (HO-1), which are antioxidant markers, were lowered by DOX and upregulated by choline. Moreover, DOX significantly decreased serum acetylcholine levels and the high-frequency component of heart rate variability and increased serum norepinephrine levels and the low-frequency component; these effects were rescued by choline administration. Interestingly, the protective effects of choline could be partially reversed by administration of the muscarinic receptor antagonist atropine. This suggests that choline might be a promising adjunct therapeutic agent to alleviate DOX-induced cardiotoxicity.

## 1. Introduction

Doxorubicin (DOX) is the most commonly used anthracycline drug against various cancers (breast cancer, Hodgkin's disease, lymphoblastic leukemia, etc.) [1, 2]. A growing stream of clinical evidences indicated that DOX has severe toxic effects on cardiac tissues, which limited the use and the therapeutic dosages of DOX [3–5]. DOX-induced cardiotoxicity has been postulated to have multiple mechanisms involving mitochondrial dysfunction, cardiomyocyte apoptosis [6–9], and increased oxidative stress and inflammation with the excessive production of reactive oxygen species (ROS) [10]. However, both ROS scavenging agents and anti-inflammatory agents, with the exception of dexrazoxane, failed to limit DOX-induced cardiotoxicity. Basic and clinical studies indicate that exposure to DOX is associated with autonomic nervous system dysfunction and cardiac dysfunction, particularly heart failure [11, 12], whereas the nonselective *β*-adrenoceptor antagonist carvedilol ameliorates DOX-induced side effects and significantly improves left ventricular function [12]. A shift in cardiac autonomic balance toward enhanced sympathetic tone occurs early during the administration of DOX-based chemotherapy, and the activated sympathetic tone results in abnormal neurohormone secretion, which is a simultaneous trigger for left ventricular dysfunction [13, 14]. Meanwhile, DOX treatment caused cardiac autonomic imbalance accompanied by elevated oxidative stress and histological changes in the heart tissue [15]. Although numerous studies have tried to understand the pathophysiology of DOX-induced cardiotoxicity, no effective treatment is available. However, increased vagal activity may represent a promising treatment strategy against DOX-induced cardiotoxicity, and it is necessary to further find effective pharmaceutical therapies for the activation of vagal tone in DOX-induced cardiotoxicity.

Choline, the precursor of the primary vagal neurotransmitter acetylcholine (Ach), confers protective benefits against numerous cardiovascular disorders, such as hypertension, myocardial infarction, ischemia/reperfusion injury, arrhythmia, and myocardial hypertrophy [16–20]. Choline exerts a remarkable cardioprotective effect by inhibiting ROS [18], preventing calcium overload [18], regulating mitochondrial dysfunction [20], and alleviating the inflammatory response [16], which are associated with improved vagal activity. Choline treatment can rebalance vagal and sympathetic activity, as evidenced by improved parameters of heart rate variability (HRV), increased serum Ach levels, and enhanced baroreflex sensitivity [16, 20]. However, it remains unclear whether choline can protect the heart against DOX-induced cardiotoxicity. In this study, we investigated the cardioprotective effects of choline in DOX-induced cardiotoxicity and explored the potential mechanisms, which offers a novel therapeutic tactic for DOX-induced cardiotoxicity.

## 2. Methods

### 2.1. Animal Preparation and Experimental Protocols

**Figure 1:**
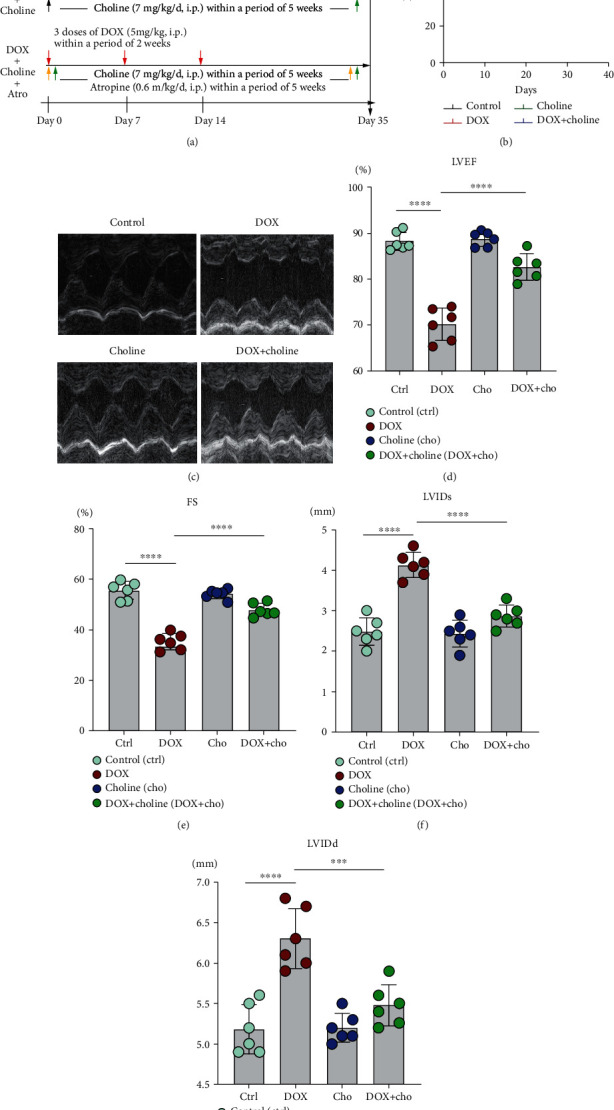
Schematic protocol for rat treatments and echocardiography. (a) Study procedure of each group. (b) The survival curves were shown (*n* = 10-15 per group). (c) Representative echocardiography images of rats from various groups. (d–g) LVEF%, FS%, LVIDs, and LVIDd were assessed by echocardiography. *n* = 6. ^∗^*P* < 0.05, ^∗∗∗^*P* < 0.001,  and^∗∗∗∗^*P* < 0.0001. LVEF%: left ventricular ejection fraction; FS: fractional shortening; LVIDs: left ventricular internal dimension in systole; LVIDd: left ventricular internal dimension in diastole.

All experimental operations were in accordance with the Guide for the Care and Use of Laboratory Animals (National Institutes of Health Publication No. 85-23, revised 1996) and were approved by the Animal Welfare & Ethics Committee of Renmin Hospital of Wuhan University (approval number: WDRM20170620). Adult male Sprague–Dawley (SD) rats weighing 180–200 g were acquired from Vital River Co. Ltd. (Beijing, China) and were fed in a 12/12 h light/dark cycle with free access to food and water. The rats were treated with doxorubicin (HY-15142, MCE) (5 mg/kg) three times on days 0, 7, and 14 via intraperitoneal (i.p.) injection. The schematic protocol for rat treatments is shown in Figure 1(a). The animals were acclimatized for 1 week and randomly divided into five groups: (1) control group—rats received only sterile saline; (2) DOX group—rats were injected with DOX (5 mg/kg) on days 0, 7, and 14 based on previous reports [14, 21]; (3) choline group—rats were injected with choline (HY-26978, MCE) (7 mg/kg/d, i.p.); (4) DOX+choline group—rats were injected with the abovementioned DOX and choline doses every day; and (5) DOX+choline+atropine (Atro)—atropine (HY-B1205) (0.6 mg/kg/d, i.p.) was injected into rats prior to the administration of choline and DOX and then concurrently with them each day. The dosages of choline and atropine were selected based on previous reports [20].

### 2.2. Echocardiography

Rats were anesthetized with isoflurane (induction 4% and thereafter 1.5%) after administration for 5 weeks. Transthoracic echocardiography was used to assess cardiac function in superficial anesthesia rats using a high-resolution imaging system (GE Vivid E95, USA, 12-MHz transducer) as previously reported [14]. The testing and analysis were conducted blinded to the experimental grouping and treatment. The left ventricle ejection fraction (LVEF%), LV fractional shortening (FS%), and LV internal diameter in systole and diastole (LVIDs and LVIDd, respectively) were assessed.

### 2.3. Measurement of Heart Rate Variability

All surface electrocardiograms were recorded to measure HRV by using a Power Lab data acquisition system (AD Instruments, New South Wales, Australia) with 1.5% isoflurane inhalation. Low-frequency (LF) power in the frequency range 0.20–0.75 Hz, which is related to the interaction of cardiac sympathetic and vagal influences, and high-frequency (HF) power in the 0.75–2.5 Hz frequency band, which is exclusively under vagal regulation, were used to test power spectrum analysis and were described in previous studies [22]. HRV's normalized LF power has been suggested to reflect sympathetic cardiac modulation, particularly when the cardiac sympathetic drive is active [23]. The LF/HF ratio was determined and used as a measure of sympathovagal balance.

### 2.4. Serum Biochemistry and ELISA Analysis

Blood was obtained from the abdominal aorta of each rat at the end of the experiment. Approximately 4 ml of blood was subjected to a standard separation procedure by centrifugation at 3000 rpm for 15 min at 4°C, and then, samples were isolated and stored at −80°C. Serum levels of creatine kinase (CK), creatine kinase-MB (CK-MB), and lactate dehydrogenase (LDH) were measured with an autonomic biochemical analyzer. Malondialdehyde (MDA), superoxide dismutase (SOD), glutathione peroxidase (GSH-PX) activity, and Ach in serum were analyzed using commercial kits following the manufacturer's protocols (Nanjing Jiancheng Bioengineering Institute, Nanjing, China).

Serum catalase (CAT), hydrogen peroxide (H_2_O_2_), glutathione (GSH), and norepinephrine (NE) were analyzed using a commercial ELISA kit (Wuhan Msk Bio Bioengineering Institute, Wuhan, China) according to the manufacturer's instructions.

### 2.5. Evaluation of Inflammatory Response

Small pieces of cardiac tissue were homogenized and centrifuged (1000 g × 10 min, 4°C), and the supernatant was collected carefully for analysis. The tumor necrosis factor-*α* (TNF-*α*), interleukin-6 (IL-6), and interleukin-1*β* (IL-1*β*) levels were analyzed using a commercial ELISA kit according to the manufacturer's instructions.

### 2.6. Evaluation of Oxidative Stress

SOD and MDA in cardiac tissue were measured using assay kits (Nanjing Jiancheng Bioengineering Institute, Nanjing, China) according to the manufacturer's protocol to evaluate the levels of oxidative stress.

### 2.7. Histological Analysis

At the end of the experiment, heart tissues from the 5 groups were isolated, washed, fixed in 4% formalin, and embedded in paraffin. Then, the tissues were sliced into 4 *μ*m thick sections and stained with hematoxylin and eosin (H&E) and Masson's trichrome. The positively stained (blue) fibrotic area was analyzed using Image-Pro Plus v.6.0. For each section, the average percentage of fibrosis to total area was determined using six random images.

### 2.8. TUNEL Staining Analysis

Cardiac apoptosis in myocardial tissue was determined by a TUNEL kit (Roche, Germany) according to the manufacturer's instructions. Apoptosis was assessed using fluorescence microscopy, and relative quantification was analyzed using Image-Pro Plus v.6.0. The ratio of TUNEL-positive nuclei to DAPI-stained nuclei in six random images per slide was used to assess apoptosis.

### 2.9. Immunofluorescence Staining

ROS production was assessed by ROS staining solution (D7008, SIGMA) in fresh-frozen myocardial sections of the left ventricle. Briefly, the frozen sections were restored to room temperature and agitated in an autofluorescence quenching solution on an orbital shaker. Next, ROS staining solution was added, and the slices were incubated at 37°C for 30 min in the dark. The nuclei were counterstained with DAPI. Finally, the slices were mounted on slides and imaged under a microscope. The fluorescence intensity in the myocardial sections was quantified with Image-Pro Plus v.6.0 as a measure of ROS.

### 2.10. Western Blotting

The heart's LV was snap frozen and lysed with RIPA solution. SDS–PAGE was used to separate the isolated protein (40 *μ*g), which was then transferred onto polyvinylidene fluoride membranes and incubated at room temperature for 1 h. Then, the membranes were incubated with primary antibody overnight at 4°C. The following primary antibodies were used: cleaved caspase 3 (c-caspase 3, 1 : 500, Cell Signaling Technology, Boston, USA), Bax (1 : 500, Abcam, Cambridge, UK), Bcl-2 (1 : 500, Abcam, Cambridge, UK), Nrf2 (1 : 500, Gene Technology, Shanghai, China), and HO-1 (1 : 200, Santa Cruz, CA, USA). After washing 4 times in TBST for 5 min, the membranes were incubated with goat anti-rabbit antibody HRP-conjugated secondary antibody (1 : 5000 to 1 : 10000; Cell Signaling Technology, Boston, USA) at room temperature for 1 h in the dark. After washing with TBST, the band intensities were analyzed by the Odyssey Imaging System (LICOR Biosciences, Lincoln, USA).

### 2.11. Statistical Analysis

All data are presented as the mean ± standard deviation (SD). Between-group differences were assessed by Student's *t*-test or one-way analysis of variance (ANOVA) as appropriate, followed by Tukey's multiple comparisons or Bonferroni post hoc test. Kaplan-Meier survival curves were constructed and compared with the log-rank test. All statistical analyses were performed using GraphPad Prism 9 software. *P* values < 0.05 were considered statistically significant.

## 3. Results

### 3.1. Choline Prevented DOX-Induced Left Ventricular Dysfunction

We recorded mortality rate in rats after DOX treatment. The results showed that the survival rate was higher in the DOX+choline group than in the DOX group (Figure 1(b)). Echocardiography was performed to evaluate the effects of choline on cardiac dysfunction under DOX administration. Compared to the control group, DOX-treated rats showed significant increases in LVIDd and LVIDs, along with decreases in LVEF% and FS% (Figures 1(c)–1(g)). In contrast, the DOX+choline group showed a substantial decrease in LVIDd and LVIDs, as well as a rise in LVEF% and FS%. It is noteworthy that rats administered choline alone did not show substantial differences compared to the control.

### 3.2. Choline Prevented DOX-Induced Cardiac Injury and Remodeling

**Figure 2:**
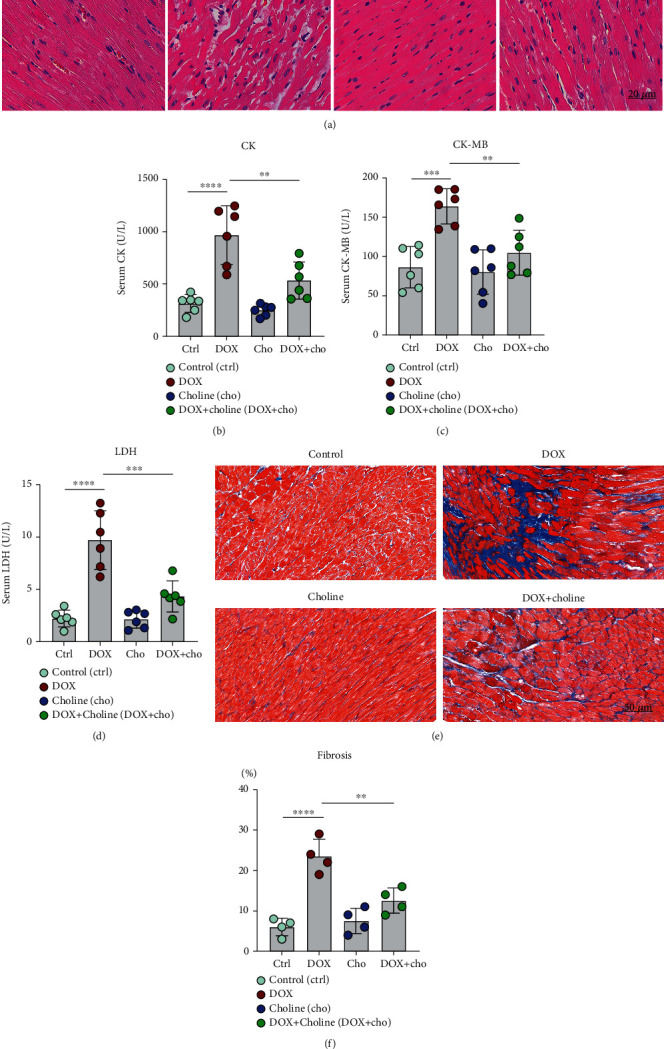
Effect of choline treatment on cardiac injury and fibrosis in DOX-treated rat hearts. (a) Typical images of H&E staining; *n* = 6; bar = 20 *μ*m. (b–d) Serum CK, CK-MB, and LDH levels were measured at the end of the experiment; *n* = 6. (e) Myocardium stained with Masson. (f) Quantitative analysis of the trichrome-positive area/total area; *n* = 4; bar = 50 *μ*m. ^∗∗^*P* < 0.01,  ^∗∗∗^*P* < 0.001, and^∗∗∗∗^*P* < 0.0001. CK: creatine kinase; CK-MB: creatine kinase myocardial isoenzyme; LDH: lactate dehydrogenase.

Histological examination showed that edema of cardiomyocytes, disordered arrangement, and inflammatory infiltrate were increased in DOX-treated rats but significantly ameliorated in the DOX+choline group (Figure 2(a)). CK, CK-MB, and LDH are the primary markers that reflect the extent of cardiac injury. Our results showed that CK, CK-MB, and LDH levels in serum were higher in the DOX group than in the control group and were attenuated by choline treatment (Figures 2(b)–2(d)). No significant difference in CK, CK-MB, or LDH levels was found between the control and choline groups. Masson's trichrome staining showed a substantial increase in interstitial fibrosis in DOX group hearts, which was attenuated by choline coadministration (Figures 2(e) and 2(f)). These results indicate that choline treatment inhibited cardiac fibrosis and improved cardiac function from DOX-induced cardiotoxicity.

### 3.3. Choline Attenuated DOX-Induced Oxidative Stress

**Figure 3:**
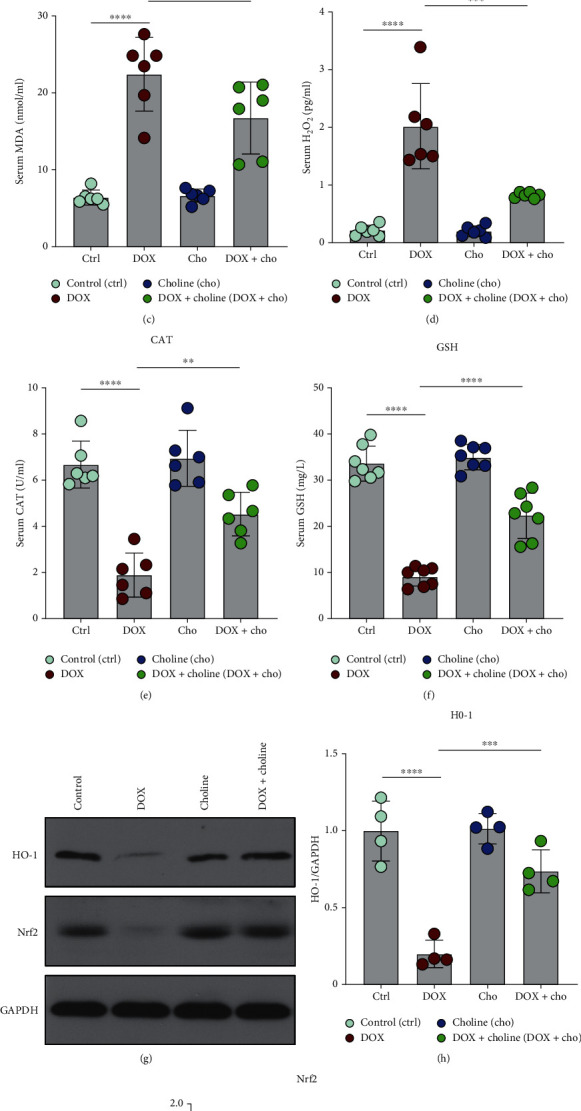
Effect of choline treatment on oxidative stress parameters. (a–f) SOD activity, GSH-PX activity, MDA level, H_2_O_2_ level, CAT activity, and GSH activity were measured; *n* = 6. (g) Western blotting bands for HO-1 and Nrf2; (h, i) Quantitative results of HO-1 and Nrf2; *n* = 4. ^∗^*P* < 0.05,  ^∗∗^*P* < 0.01,  ^∗∗∗^*P* < 0.001, and^∗∗∗∗^*P* < 0.0001. SOD: superoxide dismutase; GSH-PX: glutathione peroxidase; MDA: malondialdehyde; H_2_O_2_: hydrogen peroxide; CAT: catalase; GSH: glutathione; HO-1: heme-oxygenase-1; Nrf2: nuclear factor erythroid 2-related factor 2.

Cardiac antioxidant markers were determined by measuring SOD, GSH-Px, MDA, H_2_O_2_, CAT, and GSH content. As shown in Figures 3(a)–3(f), compared to the control group, the DOX group showed significantly decreased levels of SOD, GSH-Px, CAT, and GSH but with increased content of MDA and H_2_O_2_. In contrast, rats receiving DOX+choline showed significantly increased SOD, GSH-Px, CAT, and GSH levels and decreased MDA and H_2_O_2_ levels. We further explored the underlying mechanism of DOX-induced oxidative stress. The DOX group showed significantly lower expression levels of Nrf2 and HO-1 than the control group, while cotreatment with choline prevented this decrease (Figures 3(g)–3(i)). These results indicate that choline treatment could protect rats from DOX-induced oxidative stress damage by enhancing the antioxidant capability.

### 3.4. Choline Alleviated DOX-Induced Proinflammatory Markers and Apoptosis

**Figure 4:**
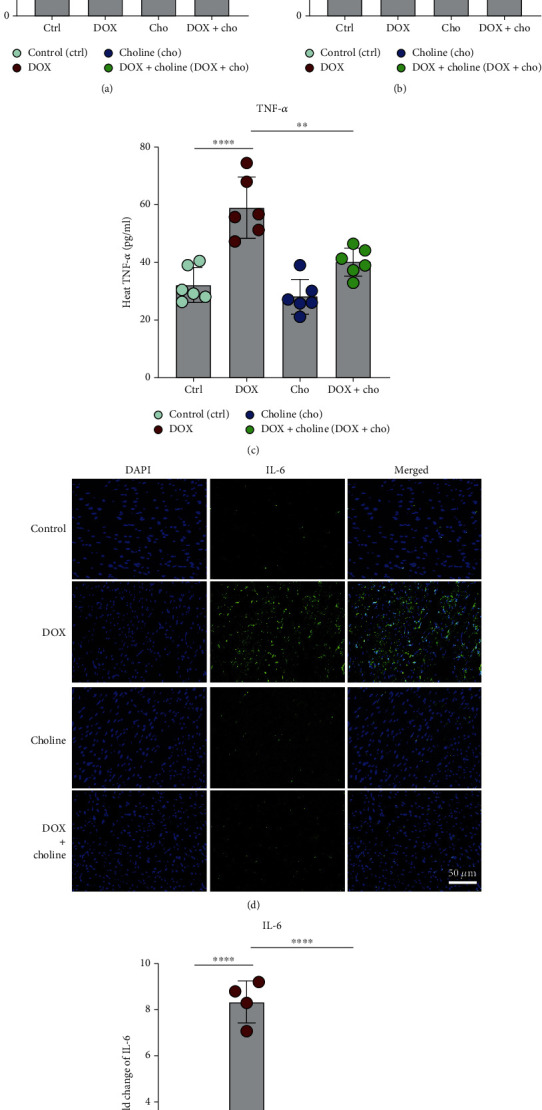
Effect of choline treatment on proinflammatory factors in cardiac tissue. (a–c) IL-6, IL-1*β*, and TNF-*α* levels were measured in cardiac tissue; *n* = 6. (d) Immunofluorescence of IL-6 in cardiac tissue; bar = 50 *μ*m. (e) Quantitative analysis of the fold change in IL-6; *n* = 4. ^∗∗^*P* < 0.01,  ^∗∗∗^*P* < 0.001, and^∗∗∗∗^*P* < 0.0001. IL-6: interleukin-6; IL-1*β*: interleukin-1*β*; TNF-*α*: tumor necrosis factor alpha.

**Figure 5:**
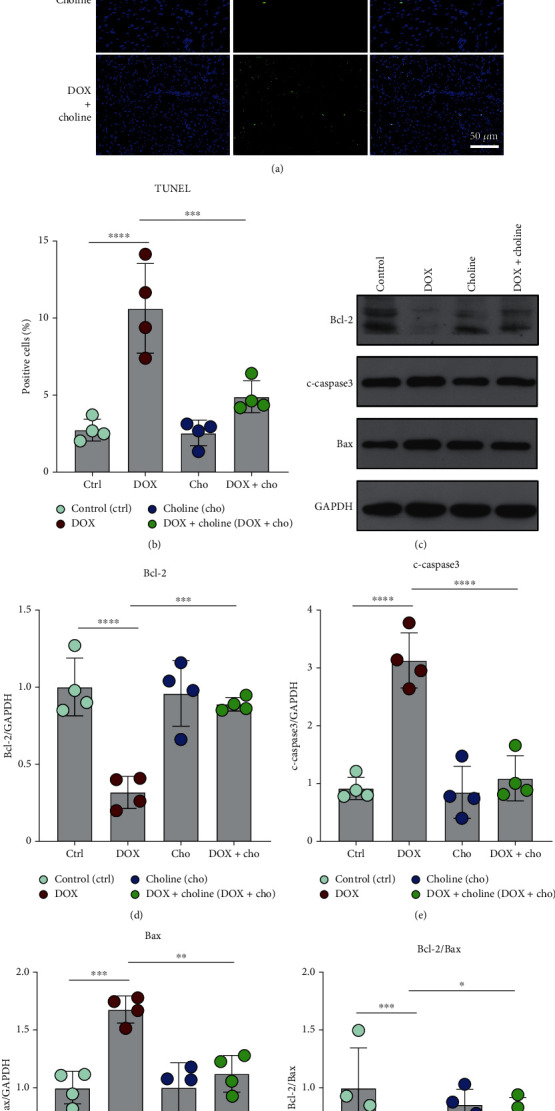
Effect of choline treatment on apoptosis in cardiac tissue. (a) TUNEL staining; bar = 50 *μ*m. (b) Quantitative analysis of the positive apoptotic cells (%); *n* = 4. (c) Western blotting bands for Bcl-2, c-caspase 3 and Bax. (d–g) Quantitative results of Bcl-2, c-caspase 3, Bax, and Bcl-2/Bax; *n* = 4. ^∗^*P* < 0.05,  ^∗∗^*P* < 0.01,  ^∗∗∗^*P* < 0.001, and^∗∗∗∗^*P* < 0.0001.

A significant increase in the expression of the inflammatory markers IL-1*β*, IL-6, and TNF-*α* in myocardial tissue was found in the DOX group compared to the control. Treatment with choline alleviated this increase compared to the DOX group (Figures 4(a)–4(c)). Additionally, compared with the control, DOX significantly increased the expression of the proinflammatory marker IL-6 in heart tissues, and this effect was attenuated by administration of choline (Figures 4(d)–4(f)). TUNEL staining was used to further assess apoptosis in myocardial tissue, which showed higher myocardial apoptosis in the DOX group than in the control and was alleviated by choline administration (Figures 5(a) and 5(b)). Apoptosis-related proteins were further investigated via Western blot analysis. The protein levels of Bax and c-caspase 3 in cardiac tissue were increased, and Bcl-2 expression was decreased in the DOX group compared to controls (Figures 5(c)–5(g)). These results showed that choline treatment alleviated myocardial inflammation and apoptosis from DOX-induced cardiotoxicity.

### 3.5. Choline Increased the Parasympathetic Tone and Reduced DOX-Induced Sympathetic Overstimulation

**Figure 6:**
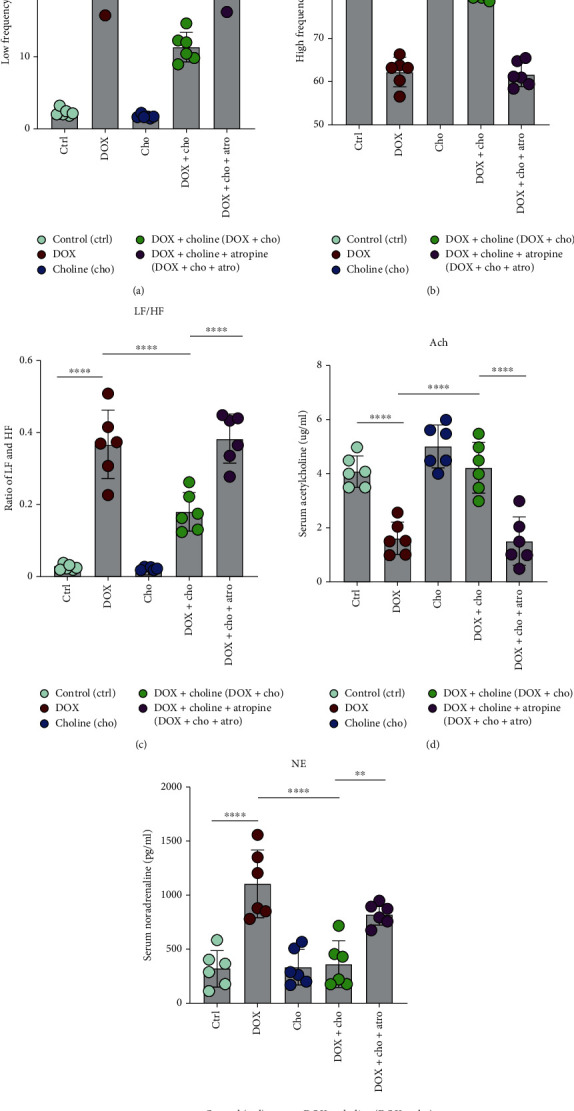
Measurement of cardiac autonomic tone. (a–c) LF power, HF power, LF/HF, and absolute values of the LF/HF ratio; *n* = 6. (d) Serum Ach level; *n* = 6. (e) Serum NE level; *n* = 6. ^∗∗^*P* < 0.01 and^∗∗∗∗^*P* < 0.0001. LF: low-frequency; HF: high-frequency; Ach: acetylcholine; NE: norepinephrine.

To further observe changes in parasympathetic and sympathetic tones with choline treatment, we used HRV. For the frequency domain, DOX significantly decreased the HF and increased the LF and the ratio of LF/HF (Figures 6(a)–6(c)), which were reversed in the DOX+choline group. There was no significant difference in the HRV frequency domain parameters between the DOX and DOX+choline+Atro groups. The Ach level in serum was decreased, and the NE level was increased by DOX administration (Figures 6(d) and 6(e)), which were attenuated by choline treatment. Atropine, a muscarinic acetylcholine receptor antagonist, eliminated the effects of choline on HRV, Ach, and NE in DOX-induced changes, suggesting that choline can activate the vagal nerve. These results indicate that DOX-treated rats exhibited high sympathetic tone and low vagal tone, while choline treatment rebalanced autonomic activity, which was prevented by atropine.

### 3.6. Activation of the Vagus Nerve Mediated the Protective Effects of Choline against DOX-Induced Cardiotoxicity

**Figure 7:**
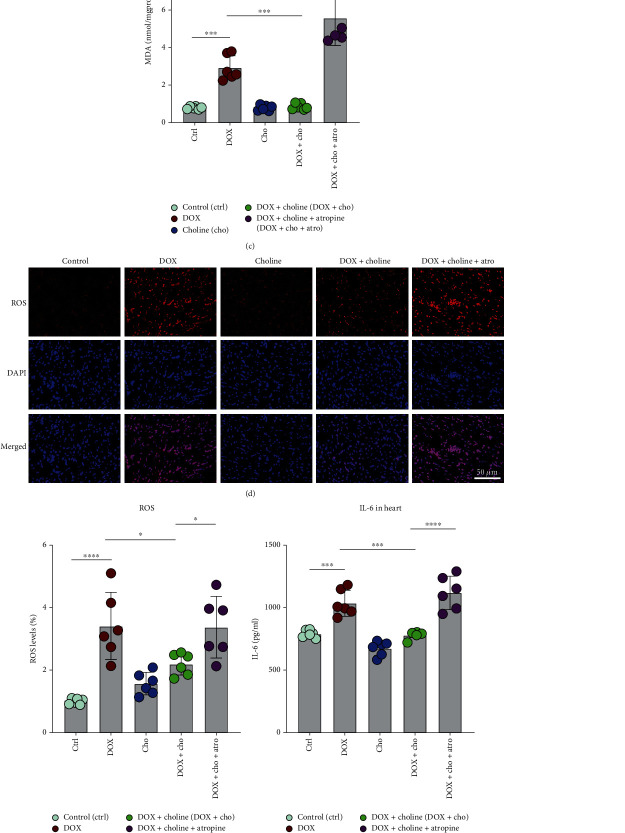
Effect of cholinergic signaling pathways on oxidative stress and inflammation. (a) Serum SOD activity. (b, c) SOD and MDA levels in cardiac tissue; *n* = 6. (d) Immunofluorescence of ROS expression in cardiac tissue; bar = 50 *μ*m. (e) Quantitative analysis of ROS expression; *n* = 6. (f) IL-6 and IL-*β* levels in cardiac tissue; *n* = 6. ^∗^*P* < 0.05,  ^∗∗^*P* < 0.01,  ^∗∗∗^*P* < 0.001, and^∗∗∗∗^*P* < 0.0001. SOD: superoxide dismutase; MDA: malondialdehyde; ROS: reactive oxygen species; IL-6: interleukin-6; IL-1*β*: interleukin-1*β*.

To further understand how the vagus nerve plays a part in choline's protective action against DOX-induced cardiotoxicity, rats were given atropine to block the Ach-activated component. We then determined the expression of the indicators for both oxidative stress and inflammation in cardiac tissue. As shown in Figures 7(a)–7(c), an increase in SOD activity and a decrease in MDA levels were observed in the DOX+choline group, while atropine significantly alleviated these changes. We also evaluated the ROS level in the myocardial tissue using immunofluorescence staining (Figure 7(d)). The increased generation of ROS was also decreased in DOX+choline hearts compared to the DOX group but was reversed when rats were treated with atropine (Figure 7(e)). We then assayed the expression of proinflammatory factors. DOX+choline hearts showed markedly lower levels of IL-6 and IL-1*β* than the DOX group, while cotreatment with atropine prevented this decrease (Figures 7(f) and 7(g)). There was no significant difference in the tested markers between the DOX and DOX+choline+Atro groups. These results indicated that choline alleviated DOX-induced cardiotoxicity through the activation of the vagus nerve.

## 4. Discussion

### 4.1. Major Findings

In the present study, we found that the combined use of choline from the early stage protected rats from DOX-induced cardiotoxicity, with improved cardiac function, inhibited inflammatory response and oxidative stress, and reduced myocardial apoptosis. Moreover, DOX-induced autonomic imbalance and sympathetic predominance were significantly reversed by choline. The protective effect of choline may be associated with the enhancement of vagal activity and the activation of the Nrf2/HO-1 signaling pathway in DOX-treated rats. Choline might be a promising adjunct therapeutic modality for the alleviation of DOX-induced cardiotoxicity (Supplemental Graphical Abstract (available here)).

### 4.2. Autonomic Imbalance Participates in DOX-Induced Cardiotoxicity

DOX, as a chemotherapeutic agent, is widely used for the chemotherapy of various cancers, including hematological malignancies, lymphoma, and many other types of solid tumors [2, 24]. It is known that DOX-induced cardiotoxicity is dose-dependent, cumulative, and progressive, which will cause irreversible damage to cardiomyocytes [24, 25]. Therefore, it is necessary to thoroughly understand the mechanism of cardiotoxicity and provide early cardioprotective interventions for patients before and during the DOX administration. Extensive basic research has revealed that oxidative stress, mitochondrial ROS production, metabolism dysregulation, iron metabolism, inflammation, calcium homeostasis dysregulation, autophagy, autonomic imbalance, and immunometabolism are possible underlying mechanisms of DOX-induced cardiotoxicity [10, 26, 27]. At present, dexrazoxane is the only agent licensed by the US Food and Drug Administration to reduce the cardiotoxicity induced by DOX [28]. Therefore, research on novel cardioprotective drugs or interventions has become an urgent priority in DOX-associated cardiotoxicity.

Previous studies have demonstrated that autonomic imbalance, such as sympathetic hyperactivity and parasympathetic hypoactivity, is associated with DOX-induced cardiotoxicity [11]. DOX has been shown to reduce rMSSD and HF power and increase LF power and the LF/HF ratio, which indicates a shift in the autonomic balance toward sympathetic predominance [11]. Similarly, clinical evidence found a significant correlation of LVEF% decreased with the LF/HF ratio and plasma norepinephrine levels [29]. The changes in DOX-induced HRV match the characteristics of the initial pathophysiological changes that occur in congestive heart failure, with a significant decrease in vagal tone and a significant increase in sympathetic tone [30, 31]. Moreover, the autonomic imbalance likely aggravated DOX-induced oxidative stress, the inflammatory response, and myocardial apoptosis and further exacerbated cardiac injury [14]. Previous studies have shown that vagus nerve stimulation suppresses myocardial apoptosis, oxidative stress, and inflammation via regulation of autonomic nerve balance [32, 33]. Our recent work also indicates that noninvasive transcutaneous vagal nerve stimulation is cardioprotective against DOX-induced autonomic imbalance and cardiac sympathetic nerve remodeling [14]. The present study is consistent with previous studies showing that DOX treatment significantly increased sympathetic activity and impaired vagal activity, as evidenced by the enhanced LF and LF/HF ratio associated with increased serum NE and decreased Ach levels.

### 4.3. Choline Ameliorates DOX-Induced Cardiotoxicity by Activating the Vagus Nerve to Correct the Autonomic Imbalance

Choline is crucial for its biological function and molecular function as a precursor of the neurotransmitter Ach. It showed a cardioprotective effect via inhibition of ROS activity and oxidative stress, restoration of intracellular calcium concentration, reduction of the levels of inflammatory factors, and enhancement of vagal activity [19, 20, 34, 35]. The latest research has shown that neuronal cholinergic pathways are conducive to the impact of choline on ameliorating cardiac hypertrophy [20]. Choline could improve HF and baroreflex sensitivity, increase serum Ach levels, and augment *Δ*HR in response to methylatropine administration in a rat heart cardiac hypertrophy model [16, 20].

A link between reduced vagal tone and the development of cardiovascular disease has been identified in many studies, and basic and clinical studies have revealed that boosting vagal activity could protect the heart [36–38]. The inhibition of vagus nerve activity has been reported in the heart tissue challenged with DOX therapy, which could contribute to cardiac dysfunction or damage [11]. Consequently, we hypothesized that choline could exert a protective effect in DOX-induced cardiotoxicity. In the present study, we found that choline treatment improved left ventricular dysfunction and attenuated cardiac injury, myocardial fibrosis, and apoptosis. Choline ameliorating DOX-induced cardiotoxicity is closely related to vagus nerve activation and sympathetic nerve inhibition, as evidenced by the enhancement of HF and serum Ach levels and the decrease in LF, the LF/HF ratio, and serum NE levels, suggesting autonomic rebalance. These data suggest that cholinergic nervous activation is involved in the cardioprotective effect of choline.

### 4.4. Choline Protects against DOX-Induced Cardiotoxicity by Activating the Vagus Nerve to Enhance Nrf2/HO-1 Signaling

Some studies have demonstrated that the Nrf2/HO-1 signaling pathway plays a central role in regulating antioxidants in cardioprotection against oxidative stress and in DOX-induced cardiotoxicity [6, 39]. Nrf2 is an important antioxidant transcription factor that regulates multiple antioxidant defense genes (e.g., HO-1, SOD, GSH, CTA, GPX, and CAT) [40]. Under oxidative stress, the Nrf2 signaling pathway is activated and leads to downstream transcription of antioxidant factors, particularly HO-1 [41]. HO-1 is a novel enzyme with powerful antioxidant and anti-inflammatory effects [42, 43]. The Nrf2/HO-1 signaling pathway can regulate oxidative stress, inflammation, and mitochondrial apoptosis [44–46]. Our previous study also suggested that one of the cardioprotective mechanisms triggered by vagus nerve stimulation is mediated by the Nrf2/HO-1 pathway [47]. In this study, the DOX-treated rats showed significantly decreased Nrf2 and HO-1 expression. In contrast, the rats cotreated with choline showed significantly higher Nrf2 and HO-1 expression. These results suggested that choline led to an increase in the expression of Nrf2 and its subsequent translocation to the nucleus, which significantly activated Nrf2/HO-1 signaling.

The Nrf2/HO-1 signaling pathway plays a key role in scavenging ROS and alleviating oxidative stress [48]. Previous studies have indicated that reducing the production of ROS is beneficial to ameliorate cardiomyopathy associated with DOX [49]. Consistent with these results, our study found that ROS production was increased under DOX administration and decreased by choline. Moreover, our results showed that DOX treatment decreased the expression of SOD, GSH-PX, GSH, and CTA compared to the control treatment. Therefore, choline treatment results in Nrf2/HO-1 activation and enhances the expression of SOD, GSH-PX, GSH, and CTA, which suggests that choline may alleviate DOX-induced cardiotoxicity through Nrf2/HO-1 redox signaling pathway activation.

Excessive oxidative stress could result in inflammatory reactions, which are also found in DOX treatment. Earlier studies suggested that DOX causes inflammatory reactions in the vasculature and myocardium and elevates the concentrations of proinflammatory cytokines (e.g., TNF-*α*, IL-1*β*, and IL-2) [50]. A critical target for anti-inflammatory action is the Nrf2/HO-1 signaling pathway [51]. In this study, DOX exposure provoked a host of proinflammatory factors, such as TNF-*α*, IL-1*β*, and IL-6; however, the inflammatory reaction was blunted by choline treatments. Notably, the effects of choline in mitigating inflammation and oxidative stress were blocked by atropine, as evidenced by the increased expression of IL-6, IL-1*β*, ROS, and MDA and decreased SOD activity in the heart tissue. This indicates that choline-mediated activation of the cholinergic system may represent a viable pharmacological intervention capable of inhibiting cardiac oxidative stress and inflammation by activating vagal activity and enhancing the Nrf2/HO-1 pathway.

DOX is capable of evocation the upregulation of the pro-apoptotic protein Bax, increasing apoptosis [52]. Interestingly, the transcription factor Nrf2 has been shown to upregulate the expression of antiapoptotic proteins [53]. Overexpression of Nrf2 can upregulate the expression of Bcl-2 and downregulate the expression of Bax, P53, and caspase 3 [54]. In the present study, we found that DOX treatment increased myocardial apoptosis, and this effect was alleviated by the administration of choline. Thus, we speculate that activation of the Nrf2/HO-1 signaling pathway may also reduce apoptosis against DOX-induced cardiotoxicity.

### 4.5. Study Limitations

The current research demonstrated the protective effect of choline against cardiotoxicity induced by a single antitumor drug, and further studies need to explore the effect of choline on cardiotoxicity caused by combination chemotherapy. Additionally, in our study, we used cancer-free rats. Further studies involving cancer models will be needed to better elucidate the complex interactions between choline, chemotherapeutic agents, the heart, and tumors and to eventually characterize the antitumor potential of choline. Furthermore, it is also interesting to investigate if choline could produce cardioprotective effects in other chemotherapeutic drug models, such as Daunorubicin, which also belongs to the anthracycline group.

## 5. Conclusions

The present study demonstrates for the first time that choline is able to blunt DOX-induced cardiotoxicity by inhibiting oxidative stress, inflammatory responses, and apoptosis in vivo via mechanisms that involve activation of the vagal nerve and elevation of the Nrf2/HO-1 signaling pathway.

## Data Availability

The datasets used and/or analyzed during this study are available from the corresponding author on reasonable request. Requests to access these datasets should be directed to Hong Jiang (hong-jiang@whu.edu.cn).

## Supplementary Materials

Supplementary Materials Supplemental Material includes a graphical abstract
