# Supplementary material for: Choline Protects the Heart from Doxorubicin-Induced Cardiotoxicity through Vagal Activation and Nrf2/HO-1 Pathway
Source: Oxid Med Cell Longev. 2022 Apr 5;2022:4740931. doi: 10.1155/2022/4740931 (PMC9005275; doi:10.1155/2022/4740931)
Supplement: Supplementary Materials — Supplemental Material includes a graphical abstract [file 4740931.f1.pdf]

Supplementary materials for Choline protects heart from doxorubicin-induced cardiotoxicity through vagal activation and Nrf2/HO-1 pathway

Authors: Fuding Guo, MD[a]†, Yueyi Wang, MD, PhD [a,] †, Jun Wang, MD[a]†, Zhihao Liu, MD[a], Yanqiu Lai, MD[a], Zhen Zhou, MD[a], Zihan Liu, MD[a], Yuyang Zhou, MD[a], Xiao Xu, MD[a], Zeyan Li, MD[a], Meng Wang, MD[a],Fu Yu, MD[a],Ruijie Hu, MD[a], Liping Zhou, MD[a], Hong Jiang, MD, PhD [a,] *

Affiliation:

[a] Department of Cardiology, Renmin Hospital of Wuhan University; Cardiac Autonomic Nervous System Research Centre of Wuhan University; Cardiovascular Research Institute, Wuhan University; Hubei Key Laboratory of Cardiology, Wuhan, China

† These authors contributed equally to this work.

***Address for correspondence:**

Hong Jiang, MD, PhD

Department of Cardiology, Renmin Hospital of Wuhan University,

No. 238 Jiefang Road, Wuhan City, Hubei Provicne 430060, P.R. China,

Tel: +86 27 88041911; Fax: +86 27 88040334,

E-mail: hong-jiang@whu.edu.cn

Supplemental Material includes:

Graphical Abstract

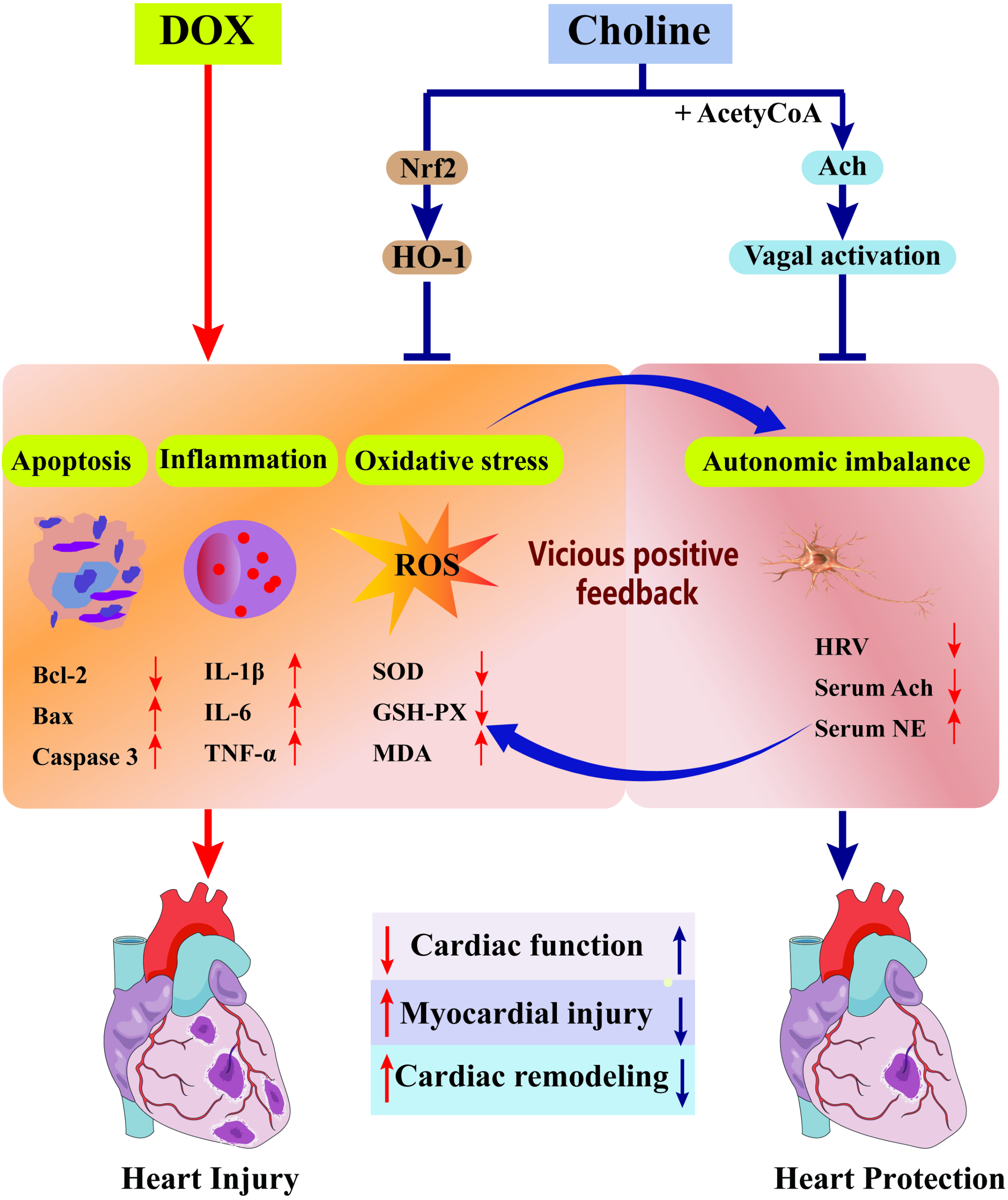

Central illustration of related mechanisms of choline in preventing DOX-induced cardiotoxicity. The protective effect of choline may be associated with the enhancement of vagal activity and the activation of the Nrf2/HO-1 signaling pathway in DOX-treated rats. A simple and non-invasive pharmacological treatment with oral choline may represent a valuable adjunct therapeutic approach for the alleviation of DOX-induced cardiotoxicity in clinic.
